# Osteolipoma independent of bone tissue: a case report

**DOI:** 10.4076/1757-1626-2-8711

**Published:** 2009-09-01

**Authors:** Bahtiyar Demiralp, Joseph F Alderete, Ozkan Kose, Ayhan Ozcan, Ilker Cicek, Mustafa Basbozkurt

**Affiliations:** Department of Orthopaedics and Traumatology, Gulhane Military Medical Academy06018 Etlik, AnkaraTurkey

## Abstract

**Introduction:**

Lipomas are the most common benign soft tissue tumors and appear in any part of the body. They typically consist of mature adipose tissue. Osteolipoma is an extremely rare histologic variant of lipoma that contains mature lamellar bone within the tumor and osteolipoma independent of bone tissue are very rare. We report a case of histologically confirmed osteolipoma independent of bone located in the thigh.

**Case presentation:**

A 47-year-old male presented with a progressively enlarging, painful mass which approximately 10 cm × 8 cm over the anteromedial aspect of his right thigh. Plain films, Computerized Tomography, Magnetic Resonance Imaging and ultrasound guided needle biopsy were performed. Given the benign imaging characteristics and fine needle aspiration, an excisional biopsy was undertaken. The definitive pathologic diagnosis was intramuscular osteolipoma without evidence of malignancy. No recurrence was observed after 18 months follow up.

**Conclusion:**

Although ossifying lipomas are very rare, it is important to keep them in mind when a lesion with adipose tissue in combination with ossification is encountered.

## Introduction

Lipomas are the most common benign soft-tissue tumors composed of only mature adipose cells without cellular atypia [1]. However, other mesenchymal elements such as smooth muscle, fibrous, chondral or osseous tissue may occasionally be found in addition to adipocytes [2]. Variants of lipoma have been named according to the type of tissue present such as fibrolipoma, myelolipoma, leiomyolipoma, chondrolipoma, osteolipoma and angiolipoma [3]. A lipoma containing mature osseous elements is called osteolipoma. The terms ossifying lipoma, osseous lipoma and lipoma with osseous metaplasia have been used interchangeably with osteolipoma [4].

Solitary lipomas are frequent, especially among adults. They can appear in any location of the body, but are usually found in the subcutaneous regions [4]. Lipoma can be located in the intraosseous region or adjacent to bone and referred to as intraosseous, parosteal, or periosteal lipoma respectively. Those that are in such sites may contain osseous and/or chondral components [5,6]. Less than 1% of lipomas were ossified in one study of 635 cases [2,4]. To our knowledge, osteolipoma independent of bone tissue has been reported in very few cases [7-10]. Most of them occurred in the head and neck area [11-18]. The differential diagnosis for an osseous containing mass in the soft tissue should cover heterotopic ossification, well-differentiated liposarcoma, osteochondroma, extra-osseous osteosarcoma, and soft tissue myxoid chondrosarcoma. Differentiating these lesions in all but heterotopic ossification, which has distinct imaging characteristics, can be very difficult and biopsy is often very helpful. We present a rare case osteolipoma independent of bone tissue located in the thigh.

## Case presentation

A 47-year old, Caucasian Turkish man presented with a progressively enlarging, painful mass over his right inguinal region which he first noticed 6 months ago. On physical examination, he had an athletic body habitus and a normal gait. He has exercised regularly for the past 27 years. A general physical examination was normal except for a firm, mobile and tender mass approximately 10 × 8 cm over the antero-medial aspect of his right thigh. It has increased slightly since its discovery. Hip and knee movements were in normal range. There were no lymph nodes palpable in the groin. No neurovascular abnormalities were found.

**Figure 1.**
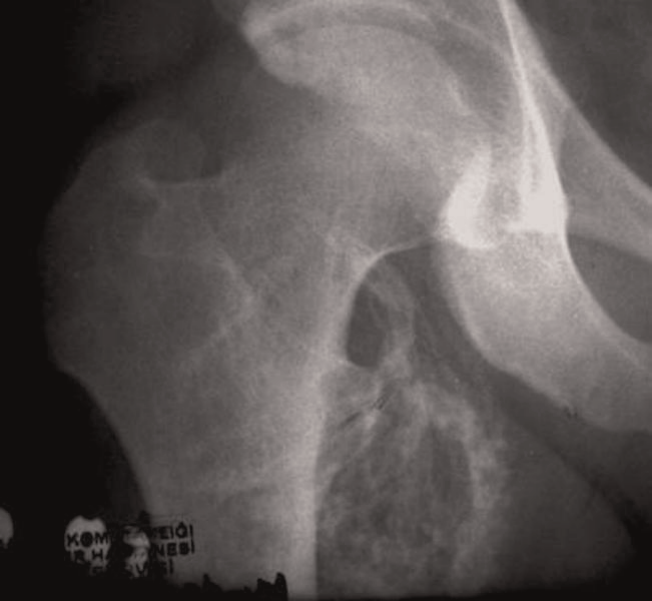
Direct radiography showing dispersed soft tissue calcifications.

**Figure 2.**
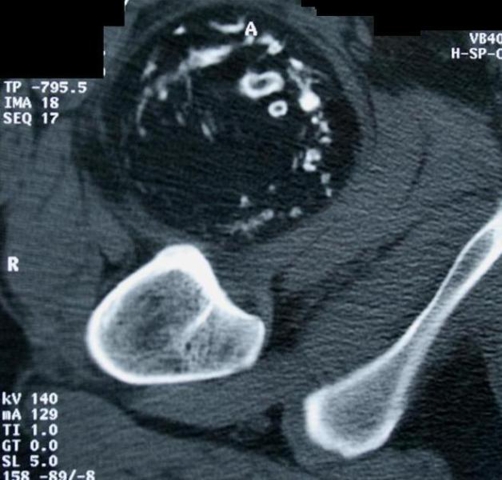
Axial CT of the right thigh showing a well-delineated, encapsulated 10 × 8 × 7 cm soft tissue mass with irregular trabeculae of bony density located in the proximal quadriceps muscle without connected to the femur.

**Figure 3.**
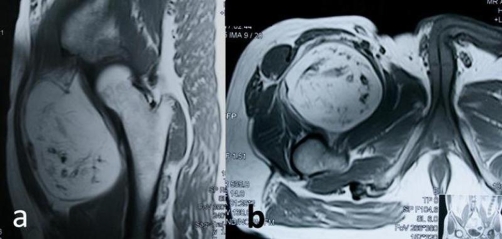
Magnetic Resonance Imaging revealed a well-circumscribed oval, lobular lipomatous mass that had high signal intensity on Tl-weighted images of the lesion. **(a)** Coronal section, **(b)** axial section.

Plain films revealed dispersed soft tissue calcifications close to the medial proximal thigh (Figure 1). Computerized Tomography showed a well-delineated, encapsulated 10 × 8 × 7 cm tumoral mass containing peripheral calcifications located in the proximal quadriceps muscle (Figure 2). Magnetic Resonance Imaging revealed a well-circumscribed oval, lobular lipomatous mass that had high signal intensity on Tl-weighted images and low signal intensity on fat-suppressed, T2-weighted images. The dense, osseous layer circumscribing the fatty core appeared as a hypo-intense cortical line on magnetic resonance imaging (Figure 3).

The pre-operative differential diagnosis was primarily a lipoma with tumoral calcinosis or an osteochondroma.

After initial imaging, ultrasound guided needle biopsy was performed revealing only benign mature fat cells and evaluated as non-diagnostic. Given the benign imaging characteristics and fine needle aspiration, an excisional biopsy was undertaken. During the operation, the tumour was found to be located in the quadriceps. At the base of the tumour a pedicle with several blood vessels was attached. Neither the tumour nor its pedicle showed any connection to bony structures. It was removed surgically by blunt dissection and the pedicle was tied and excised. We performed a multi-layered closure and the postoperative course was uneventful.

Grossly, the resected specimen consisted of a mass measuring 10 × 8 × 7 cm, with a smooth surface. Cut sections of the mass revealed mainly yellow soft tissue with numerous interlacing thin lamellar bony structures and surrounded with a thin fibrous capsule.

**Figure 4.**
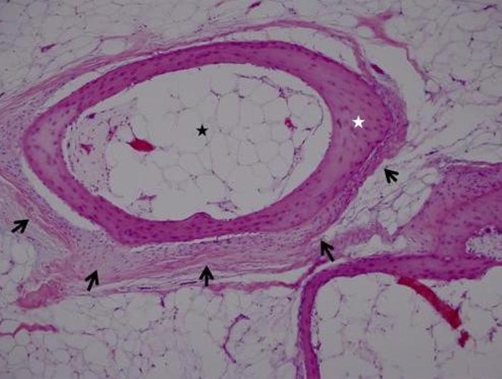
The tumor consisted largely of mature adipose tissue (black star) with surrounded by a thin layer vascularized fibrous tissue (black arrows). There was mature trabecular bone (white star) within the adipose tissue. (Hematoxylin & Eosin 100× magnification).

Histological examination revealed that the tumour consisted largely of mature adipose tissue with surrounded by a thin layer vascularized fibrous tissue. A haphazardly distributed and interlacing thin vital lamellar bone structures were found throughout the tumour. In some areas, these bony structures were alongside the thin fibrous tissue with myxoid degeneration. No myeloid cell islands were determined. The adipocytes were uniform in size and shape. No nuclear atypia, hypercellularity, mitosis nor necrosis was seen (Figure 4). The definitive pathologic diagnosis was intramuscular osteolipoma without evidence of malignancy. No recurrence was observed after 18 months follow up.

## Discussion

Osteolipoma have been reported in adult patients usually after long indolent courses. These tumors have origin from the deep soft tissue as well as from the subcutaneous plane. Osteolipoma are well encapsulated and have a sharp transition between bone and adipose tissue [1-20].

Many cases of lipomatous lesions with osseos tissue have been reported in the literature. Most of them connected with bone (inside a bone or adjacent to bone) [4,6,9]. They are intraosseous lipoma, parosteal or periosteal lipoma. Only few osteolipoma cases of without bone connection are reported in the literature [2,4,7,13,19].

The pathogenesis of osteolipoma is still not clear. Two main theories of the pathogenesis of ossifying lipomas exist. First these tumors may originate directly from multipotent mesenchymal cells since osteolipoma resembles a benign mesenchymoma [4,20]. Alternatively it has been suggested to arise after repetitive trauma, metabolic changes, or possibly ischaemia, leading to metaplasia of pre-existing fibrous elements within the lipoma and development into osteoblasts [2,13]. Our histological findings and the indolent nature of the tumour presented here support the second hypothesis. Furthermore, huge lipomas showing fast enlargement may have cystic degeneration and necrosis. Consequently, necrotic tissue may ossify mimicking osteolipoma. Conversely, most of the reported osteolipoma are very small in size [14,15].

Imaging features of osteolipoma are often pathognomonic but depend on the location of the lesion. Plain film X-rays are obtained first and yield a differential diagnosis of osteochondroma, lipoma with tumoral calcinosis, and myositis ossificans [4]. On Computerized Tomography scan, the tumour is well defined and homogeneous, having identical tissue attenuation with the surrounding normal fat. Findings of hypodense areas of fat with surrounding hyperdense layers of calcification on computerized tomography should cause one to suspect osteolipoma [12]. However, similar findings may also be seen in calcified lipoma, ossifying fibroma, osteoma, enchondroma, as well as chondroblastoma or osteochondroma on computerized tomography. It is important to obtain images with fat suppression to differentiate fat from other soft tissues on magnetic resonance imaging [12]. On Magnetic Resonance Imaging, a discrete, encapsulated, homogeneous fatty mass with similar signal intensity of the subcutaneous fat in all pulse sequences is most certainly a simple lipoma. However, lipoma variants have unusual features on imaging studies. Intralesional non-adipose components can confound the correct imaging diagnosis because they can mimic findings associated with well-differentiated liposarcomas [5].

A differential diagnosis suggesting osteolipoma primarily depends on its location. Because of the various anatomic sites reported for this lesion, a very wide range of lesions can be included in the differential diagnosis, such as other benign tumors that may contain bone including teratoma or dermoid [12]. In addition, tumor calcinosis, ossifying fibroma, hemangioma, calcified bursa and myositis ossificans should also be considered [2,4,12]. Soft-tissue sarcomas that can show calcification or ossification include liposarcoma, synovial sarcoma, extraskeletal osteosarcoma and extraskeletal chondrosarcoma. Soft-tissue chondromas, which are also rare, are frequently mineralized. Also in some series showed that osteolipoma mimicked well differentiated liposarcoma [2].

Angiolipoma, osteolipoma, and leiomyolipoma are benign processes but are often hard to differentiate from low grade liposarcoma on imaging alone, thus we recommend Computerized Tomography guided biopsy. Ultrasound fine needle aspiration is less useful given the solid nature of these tumors. If imaging is more suggestive of osteolipoma, serial magnetic resonance imaging studies are recommended so that a low grade liposarcoma is not ignored.

Definitive diagnosis of osteolipoma can easily be done with histopathologic examination. A histopathologic appearance of diffuse, mature ossification within fatty tissue clinches the diagnosis [12]. The adipose component is usually predominant and the mature bone tissue is irregular in distribution. Bone spicules are surrounded by fibrous tissue bands [2,4,8].

## Conclusion

In conclusion, osteolipoma with independent bone is very rare. It has a characteristic radiological and pathological appearance. Osteolipoma has a same prognosis as simple lipoma and surgical excision is the recommended treatment. No recurrences have been reported.

Although ossifying lipomas are very rare, it is important to keep them in mind when a lesion with adipose tissue in combination with ossification is encountered.
